# Generous hosts: Why the larvae of greater wax moth, *Galleria mellonella* is a perfect infectious host model?

**DOI:** 10.1080/21505594.2018.1454172

**Published:** 2018-05-04

**Authors:** Nabil Killiny

**Affiliations:** Citrus Research and Education Center, Department of Plant Pathology, IFAS, University of Florida, Lake Alfred, Florida, United States of America

**Keywords:** *Galleria mellonella*, GC-MS, chemical composition, haemolymph, infectious host model

The larvae of the greater wax moth, *Galleria mellonella* (Lepidoptera: Pyralidae) has been used as a host and model system for many fungal and bacterial pathogens due to its reduced innate immune system [1] and ease of handling and rearing. Organisms of interest can be injected into the haemolymph of *Galleria* where they can be maintained for up to 10 days *in vivo* and then recovered for purification or enumeration. However, in most virulence studies involving *G. mellonella*, pathogens are injected to determine their LD_50_ and/or to monitor production of antimicrobial peptides (AMPs) such as defensin and lysozyme [2,3]. Larval health after infection with various agents is evaluated by activity, cocoon formation, melanization and survival rate [4].

The insect immune system is relatively more advanced than other invertebrates such as nematodes and thus can give more insights about mammalian infection processes [5]. The *G. mellonella* immune system consists of the cellular and the humoral response [1]. The cellular response to invading pathogens is a direct response by haemocytes which surround and immobilize them by phagocytosis [2]. The humoral response consists of melanization, and production of opsonins and AMPs. The opsonin apolipophorin-III, which shows high affinity for bacterial lipopolysaccharide in insects, has high homology with mammalian apolipoprotein [1]. *G. mellonella* haemolymph contains antimicrobial peptides (gallerimycin and galiomicin) which act to break down the cell walls of fungal or bacterial pathogens [2]. Melanization synthesis and deposition serves to encapsulate pathogens at the wound site in insects [1].

*G. mellonella* has been used as a host model to study a variety of bacteria including Gram-positive bacteria such as *Enterococus faecalis*, *Enterococcus faecium*, *Staphylococcus aureus*, *Streptococcus pyogenes*, *S. pneumonia*, and *Listeria monocytogenes*; and Gram-negative bacteria such as *Escherichia coli*, *Pseudomonas aeruginosa*, and *Klebsiella pneumonia* [1]. In addition, *G. mellonella* larvae have been used to study several pathogenic fungi such as *Candida albicans* and *Aspergillus fumigatus* [6] and as a model host for some trans-kingdom pathogens such as *Fusarium oxysporum* and *Pseudomonas aeruginosa* [7]. The *G. mellonella* model was also used to assess the efficacy of bacteriophage therapy to treat pathogenic infections [8].

Its readiness to infection and its ability to initiate a defense response makes *G. mellonella* an excellent infection model [9]. Although many studies have been conducted to investigate the components of *G. mellonella* innate immune response system, the haemolymph of this interesting model has not been well investigated. To date, there are very few reports of the chemical composition of the primary and secondary metabolites of the haemolymph of wax worm [10–13] and even fewer utilizing modern GC-MS techniques. A study of the changes in amino acid (AA) composition of the haemolymph in response to temperature acclimation found 14 different amino acids by liquid chromatography and showed an increase in AA content with decreasing temperatures [13]. Earlier works focused on the lipid and lipoprotein fractions of the haemolymph by solvent extraction followed by GC-FID, and by centrifugation followed by thin layer chromatography [12,14], respectively. However, to our knowledge, no general characterization of the polar metabolites of *G. mellonella* haemolymph has been carried out which would help define its ability to host a wide range of fungal and bacterial pathogenic species.

Untargeted metabolomic profiling is extremely useful for simultaneous analysis of a broad range of metabolites within a cell type, tissue, or even whole organisms, including amino acids, organic acids, sugars, sugar alcohols and fatty acids. Analysis of small molecules which are often metabolic pathway intermediates can help identify subtle changes in complex metabolic systems and can be used comparatively. These studies can be achieved through a variety of analytical tools including liquid and gas chromatography, as well as by using different chemical derivatization techniques. Each method opens a small window of understanding into a particular metabolic pathway or the biochemistry underlying a particular condition or treatment effect. In the case of this study, we used three methods of derivatization 1) trimethylsilylation (TMS); 2) methyl chloroformate (MCF); and 3) boron trifluoride (BF3) to detect several classes of metabolites found in the haemolymph of *G. mellonella* for the purpose of ascertaining why it is useful as an infectious disease host.

**Figure 1.**
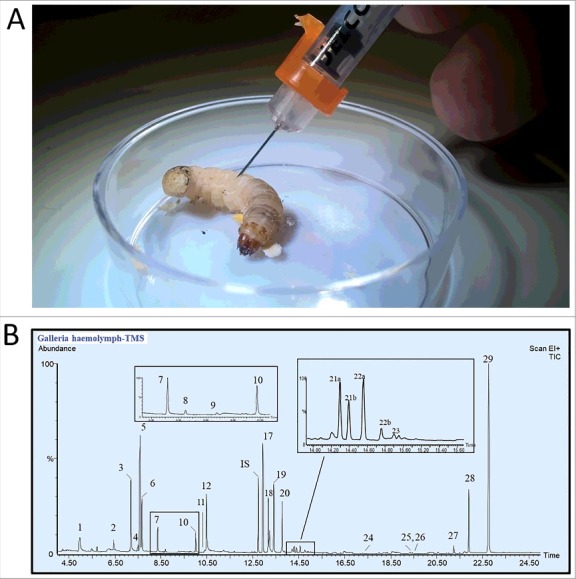
Collection and chemical analyses of *Galleria mellonella*. A: Haemolymph was collected by first puncturing the larvae behind the first set of thoracic (true) legs, in the inter-spiracle space, with a 0.5 mL BD fine insulin syringe. For each replicate, haemolymph from five larvae was collected. From 5 to 7 µL of pure haemolymph was collected from each larvae using a 10 µL capillary tube and was ejected into a 0.2 ml tube containing 50 µL of solvent (8:1:1 methanol:chloroform:water) to prevent melanization. Portions from the replicates were derivatized using TMS, MCF, or BF3 prior to the GC-MS analysis. B: GC-MS total ion chromatogram of *G. mellonela* haemolymph after TMS derivatization. The numbers above the peaks refer to the numbers listed in Table 1 Is: internal standard used to calculate the absolute amount of each compounds.

*Galleria mellonella* larvae (waxworms) were received in sawdust, from Bassett's Cricket Ranch (Visalia, CA). Waxworm larvae were fed overnight on a mixture of wheat bran, honey and water, to reverse any dehydration and starvation effects from shipping. The following day, haemolymph was collected (Figure 1A). The haemolymph was subject to the derivatization and GC-MS analyses (Figure 1B). GC conditions and methods, derivatization, compound identification and quantification for insect haemolymph were performed as described in previously published protocols [15].

Twenty-nine and 31 metabolites were detected in the haemolymph of *G. mellonella* after chemical derivatization with TMS or MCF, respectively, followed by GC-MS analysis. Tables 1 and 2 show the distribution of metabolites detected by the first two methods. Fatty acid results from the boron BF3 experiments were included in Table 2. The mean total metabolites concentration constituted 156.2 ± 74.3 mM by TMS and 108.9 ± 25.6 mM by MCF derivatization.

**Table 1.** Concentration (mM) of metabolites of *Galleria mellonella* haemolymph derivatized by trimethylsilylation (TMS) and analyzed by GC-MS. Means ± standard deviation shown were generated from total ion chromatograms (TIC) peaks from duplicate injections from five biological samples (*n* = 10). Peak areas were converted to mM concentration by use of linear calibration curves of standard reference compounds treated the same as samples.

Peak No.	LRI	TMS Derivative	Mean ± SD	[%] mM
1	1085	_L_-Alanine^a^	1.24 ± 0.63	0.79
2	1223	_L_-Valine^a^	1.15 ± 0.62	0.74
3	1278	Phosphoric acid	6.65 ± 2.43	4.25
4	1296	_L_-Isoleucine^a^	0.74 ± 0.36	0.47
5	1300	_L_-Proline^a^	15.2 ± 9.39	9.72
6	1308	Glycine^a^	4.80 ± 2.34	3.07
7	1369	_L_-Serine^a^	3.45 ± 3.13	2.21
8	1395	_L_-Threonine^a^	0.37 ± 0.06	0.23
9	1447	β-Alanine^a^	0.24 ± 0.08	0.15
10	1515	Malic acid^a^	1.47 ± 0.80	0.94
11	1538	_L_-Aspartic acid^a^	0.27 ± 0.02	0.17
12	1541	Pyroglutamic acid^a^	5.43 ± 2.34	3.47
13	1580	2-Ketoglutaric acid^a^	0.92 ± 0.15	0.59
14	1637	_L_-Glutamic acid^a^	0.16 ± 0.00	0.10
15	1641	_L_-Phenylalanine^a^	0.22 ± 0.03	0.14
16	1705	_L_-Asparagine^a^	0.41 ± 0.08	0.27
17	1752	Putrescine^a^	88.7 ± 41.8	56.67
18	1771	α-Glycerophosphate^a^	3.38 ± 0.98	2.16
19	1786	o-Ethanolamine phosphate^b^	2.75 ± 2.80	1.76
20	1819	Citric acid^a^	5.03 ± 1.33	3.21
21	1894;1906	Fructose^a^	0.91 ± 0.18	0.58
22	1916;1929	Glucose^a^	0.88 ± 0.25	0.56
23	1945	Glucitol^a^	0.22 ± 0.02	0.14
24	2013	Glucaric acid^a^	0.09 ± 0.04	0.06
25	2484	Unknown Disaccharide 1^b^	0.10 ± 0.01	0.06
26	2502	Unknown – *m/z* 456/513	0.31 ± 0.13	0.20
27	2545	Unknown Disaccharide 2^b^	0.20 ± 0.04	0.13
28	2577	Sucrose^a^	0.36 ± 0.09	0.23
29	2651	Trehalose^a^	10.8 ± 4.20	6.92
		**Total**	**156.5** ± **74.3**	**100.00**

a Compounds were confirmed using derivatized reference substances.

b Compounds tentatively identified using mass spectral databases (NIST 2011, Wiley 9^th^ Ed.) or online Golm Metabolome Database (http://gmd.mpimp-golm.mpg.de/).

**Table 2.** Concentration (mM) of metabolites of *Galleria mellonella* haemolymph derivatized by methyl chloroformate (MCF) and analyzed by GC-MS. Means ± standard deviation shown were generated from total ion chromatograms (TIC) peaks from duplicate injections from five biological samples (*n* = 10). Peak areas were converted to mM concentration by use of linear calibration curves of standard reference compounds treated the same as samples.

Peak No.	LRI	MCF Derivative	Mean ± SD	[%] mM
1	603	Fumaric acid^a^	0.22 ± 0.02	0.20
2	608	Maleic acid^a^	0.22 ± 0.02	0.20
3	614	Succinic acid^a^	0.69 ± 0.08	0.63
4	1116	Glycine^a^	5.16 ± 0.93	4.74
5	1116	_L_-Alanine^a^	10.4 ± 1.76	9.52
6	1286	_L_-Valine^a^	7.17 ± 1.40	6.59
7	1382	_L_-Leucine^a^	5.34 ± 1.21	4.91
8	1397	_L_-Isoleucine^a^	2.50 ± 0.57	2.30
9	1406	_L_-Threonine^a^	0.41 ± 0.12	0.38
10	1423	Malic acid^a^	1.43 ± 0.21	1.31
11	1433	_L_-Proline^a^	32.0 ± 4.42	29.85
12	1440	_L_-Asparagine^a^	2.71 ± 1.28	2.49
13	1489	Quinic acid^a^	5.12 ± 2.86	4.70
14	1514	_L_-Aspartic acid^a^	0.34 ± 0.24	0.31
15	1527	Citric acid^a^	7.05 ± 2.06	6.48
16	1584	_L_-Serine^a^	5.58 ± 1.38	5.13
17	1627	_L_-Glutamine^a^	7.58 ± 2.68	6.96
18	1637	_L_-Glutamic acid^a^	0.49 ± 0.30	0.45
19	1650	_L_-Methionine^a^	0.21 ± 0.06	0.19
20	1733	_l_-Cysteine^a^	0.01 ± 0.01	0.01
21	1755	_L_-Phenylalanine^a^	1.58 ± 0.35	1.45
22	1765	Myristic acid (C14:0)^a^^,^^c^	0.05 ± 0.02	0.05
23	1850	Pentadecanoic acid (C15:0)^b^^,^^c^	0.01 ± 0.01	0.01
24	1942	Palmitic acid (C16:0)^a^^,^^c^	0.57 ± 0.30	0.52
25	2009	_L_-Lysine^a^	5.43 ± 1.36	4.99
26	2054	_L_-Histidine^a^	1.03 ± 0.26	0.95
27	2072	Linoleic acid (C18:2)^a^^,^^c^	0.32 ± 0.15	0.29
28	2078	Oleic acid (C18:1)^a^^,^^c^	0.22 ± 0.06	0.20
29	2097	Stearic acid (C18:0)^a^^,^^c^	0.50 ± 0.38	0.46
30	2133	_L_-Tyrosine^a^	3.35 ± 0.69	3.08
31	2266	_L_-Tryptophan^a^	0.71 ± 0.45	0.65
		**Total**	**108.9** ± **25.6**	**100.00**

a Compounds were confirmed using derivatized reference substances.

b Compounds tentatively identified using mass spectral databases (NIST 2011, Wiley 9^th^ Ed.).

c Results from boron trifluoride derivatization.

Several non-proteinogenic amino acids were detected in the haemolymph of *G. mellonella*. Putrescine (1, 4-butadiamine) was detected only by TMS and was found to be 88.7±41.8 mM, the highest of any compound in the haemolymph of *G. mellonella*. Putrescine has also been found in the haemolymph of the corn earworm, *Heliothis zea*, at 375±25 µg/mL haemolymph by ninhydrin assay and thin layer chromatography [16]. The primary role of putrescine is binding of ammonia waste for transport prior to excretion, as was found in other arthropods [17,18]. Other non-proteinogenic amines detected by TMS included pyroglutamic acid and β-alanine (Table 1). These metabolites were not detected by MCF. Interestingly, gamma-aminobutyric acid (GABA) was detected by HPLC in larvae just before metamorphosing into adults [13], but we did not detect it in any of our analyses. This may have been due to differences in the age or storage conditions of our larvae as we routinely detect GABA in other biological samples using TMS derivatization [15].

Among the proteinogenic amino acids, proline was the major amino acid detected by both methods, TMS (15.2±9.4 mM) and MCF (32.5±4.4 mM). Proline has been implicated in hymenopterans as an alternate energy source for flight muscles [19], but its role in waxworm larvae may be more related to muscle movements associated with the wandering stage. In tarantula spider haemolymph, proline was also found to be the major amino acid [20]. Glycine and serine made up 3.1 and 2.2% of the haemolymph composition, respectively for TMS-derivatized samples, while these were 4.7 and 5.1% of the composition, respectively by MCF. Other amino acids found by both methods included alanine, valine, glutamic acid, asparagine, threonine, aspartic acid, phenylalanine and isoleucine (Tables 1 & 2). For TMS method, other amino acids detected at low concentration included alanine, valine, isoleucine, threonine, glutamic acid, phenylalanine, asparagine, and aspartic acid. These ranged in concentration from < 0.2 mM to 1.25 mM. For MCF method, several other amino acids were found in significant concentrations including lysine, histidine, and tyrosine (Table 2). Interestingly, Grace's insect medium includes 16 mM of histidine, but only lesser amounts of most other amino acids (between 0.5 and 5 mM) (Cat#11595, Thermofisher Scientific, Waltham, PA). Compounds detected at low concentration (<1 mM) by MCF included tryptophan, glutamic acid, threonine, aspartic acid, methionine and cysteine. These were not detected by TMS derivatization. Hanzal and Jegorov [13] reported that glutamine, alanine and glycine were the most abundant amino acids of control *G. mellonella* larvae, and levels of phenylalanine and lysine increased during cold acclimation of wax worms. In the same study, levels of GABA and valine increased with larval age, while glycine, alanine and glutamine decreased with increasing age [13].

Citric and malic acids were the only two organic acids detected in *G. mellonella* haemolymph by TMS and these were also found in our study of the haemolymph of the Asian citrus psyllid, *D. citri* [15]. The concentrations of citric and malic acids were 5.0 and 1.5 mM, respectively. In addition to citric and malic acids, quinic acid, and succinic acid were detected by MCF at levels of 5.1 mM and 0.7 mM respectively. Fumaric and maleic acids were also found in trace amounts, and these participate in the citric acid cycle. Grace's insect medium supplies malic acid at 5 mM, and fumaric and succinic acids at a concentration of 0.5 mM indicating that they are required nutritional elements for insects.

Numerous saturated fatty acids (FAs) were detected by BF3 including tetradecanoic (myristic) acid, pentadecanoic acid, hexadecanoic (palmitic) acid, and octadecanoic (stearic) acid. Unsaturated FAs detected were oleic (C18:1) and linoleic (C18:2). However, the relative abundance of free fatty acids was low in our larvae (1.67 mM, 0.36%) compared to Thomas (1979) [12] who found 9.3% of the whole haemolymph of *G. mellonella* was composed of fatty acids. The distribution of fatty acids was similar to that reported by Yendol (1970) [14] except that we also found C14 and C15 saturated FAs, but did not find linoelaidic acid (C18:3) or palmitoleic (C16:1). Tarantula haemolymph was similar in its FA content, consisting mostly of C16 and C18 saturated FAs with traces of C14 and C20 FAs [20]. FAs play a variety of roles in insect metabolism, especially in energy storage and demand [21], immunity [22], cell membrane structures [23] and hormone biosynthesis [12].

Trehalose, a glucose-glucose disaccharide, was the most abundant carbohydrate detected in waxworm haemolymph (Table 1), followed by the monosaccharides fructose and glucose. Trehalose constituted about 7% of the peak area while all other sugars were less than 1% each and did not exceed 9% of the overall haemolymph chemical composition. In several lepidopterans, including *G. mellonella*, trehalose was the major blood sugar, up to 90% [11]. Assuming our unknown disaccharides are truly sugars, trehalose accounts for 79.7% of the sugars found in our wax worm larvae haemolymph by TMS/GC-MS. Trehalose is commonly found in the haemolymph of many insects and serves several physiological functions including carbon storage [24], direct utilization for glucose energy metabolism [11], as a cryo-protectant [25,26], and for lowering osmotic pressure since large amounts can be stored in the fat body without toxic effects [27,28]. Levels of trehalose can vary dramatically by species, and based on ambient temperatures, activity, growth stage, and nutrition [28]. In aphids, for example, 196 mM and 926 mM trehalose were reported for the chestnut aphid, *Lachnus tropicalis* and *Aphis gossypii* respectively [29]. Recently, structural roles for trehalose were defined after RNA interference of trehalase enzymes showed deformities in chitin synthesis in larva of Colorado potato beetles [30].

We detected both inorganic and organic phosphate (Table 1) in the haemolymph of *G. mellonella*. Inorganic phosphate was quite high, about 6.7 mM, while the sugar phosphates, including glycerophosphate and ethanolamine phosphate, made up 8.2% of the haemolymph composition. The early work of Wyatt et al. (1956) in haemolymph characterization showed that inorganic phosphate found in the haemolymphs of *Bombyx mori* and *G. mellonella* ranged from 5 to 15 mg per 100 mL. Organic phosphate was reported to be 100–200 mg per 100 mL [10]. Two sugar acids, 2-ketoglutaric acid and glucaric acid, were detected, as well. The former is an intermediate in the citric acid cycle, while the latter is an acidified side-product of glucose. Not surprisingly, Grace's insect medium supplies 2-ketoglutaric acid at a concentration of 2.5 mM (Cat# 11595, ThermoFisher Scientific, Waltham, MA). Finally, glucitol, a sugar alcohol, was detected in minor concentration (0.22 mM).

In summary, by compound class, TMS derivatization detected a composition of 60.1% non-proteinogenic amino acids; 18.1% amino acids; 8.2% phosphates; 4.2% organic acids; mono- and disaccharides; 0.7% sugar acids; and the sugar alcohol, glucitol, was 0.14%. Methyl chloroformate derivatization, which favors carboxylic compounds, detected metabolites from three metabolite classes: amino acids (92.5%), organic acids (14.7%), and fatty acids (1.7%). The knowledge of chemical composition of *G. mellonella* haemolymph sheds light why G. mellonella is a good infectious host model for many microorganisms. In addition to the reduced innate immune system, *G. mellonella* haemolymph provide these pathogens with the essentials nutrients needed to multiply.
